
JOURNAL INFORMATION
==============================
NLM Title Abbreviation: Atlanta Med Surg J (1884)
NLM Journal ID: 101251623

Atlanta Medical and Surgical Journal (1884)

Publisher: Biomedical Journal Digitization Project

ARTICLE INFORMATION
==============================
PMCID: PMC9201847
PMID: 35829603
Article version: 1
Subjects: Original Communications

The Southern Surgical and Gynecological Association

Proceedings of the Eleventh Annual Meeting, Held at Memphis, Tennessee, December 6, 7, and 8, 1898

Print publication date: 1899 Jan
Volume: 15
Volume Series: New Ser
Issue: 11
First page: 725
Last page: 742
License: This work is free of known copyright restrictions. For more information, please see PMC Back Issue Digitization.
License URL: https://creativecommons.org/publicdomain/mark/1.0/

==============================

inserted and tied, the peritoneum will now be turned in and the stitches hidden.

The operation having been completed, and the dressings applied, the patient is placed between the folds of a warm blanket and only a little warm water allowed by mouth for the first twelve or eighteen hours. Then a few drachm doses of liquid peptonoids are given at intervals of twenty minutes, and, if well borne, peptonized milk is added. The tendency is to give too small quantities of food at too frequent intervals. The bowels are moved on the third or fourth day by small doses of calomel and soda.

In closing Dr. Wiggin laid great emphasis on the fact, that the prognosis in this class of cases depends more upon the promptness with which surgical treatment is instituted than upon any other factor.

---

### THE SOUTHERN SURGICAL AND GYNECOLOGICAL ASSOCIATION.

*Proceedings of the Eleventh Annual Meeting, Held at Memphis, Tennessee, December 6, 7, and 8, 1898.*

The Association met at the Gayoso Hotel, under the presidency of Dr. Richard Douglas, of Nashville, Tenn.

Prayer was offered by Bishop Gailor.

Dr. Alexander Erskine, of Memphis, delivered an "Address of Welcome" on behalf of the local profession, which was responded to by President Douglas.

After the disposal of routine business, the scientific session was opened by a paper by Dr. William E. Parker, of New Orleans, La., entitled:

### GUNSHOT WOUNDS.

He described the hospital conditions in Cuba and the wounds seen by him made by the modern bullet. He spoke of some experiments, made under the direction of Major and Surgeon L. A. LaGarde, of the regular army, as to the relative penetrating powers of the Mauser, Krag-Jorgensen and Springfield rifles.

Dr. F. W. McRae, of Atlanta, followed with a paper on

PENETRATING WOUNDS OF THE ABDOMEN, WITH REPORTS OF CASES.

At the outset, the essayist referred to a resolution passed unanimously by the Association at its Nashville meeting in 1896, to the effect that in all cases of penetrating wounds of the abdomen it is the duty of the surgeon to make an exploratory incision so as to definitely determine whether the viscera have been injured or not. Since then a few able surgeons have dissented from this opinion. The propriety of surgical interference in cases of penetrating gunshot wounds of the abdomen will depend on one of three things: (1) The general condition of the patient, (2) dangerous internal hemorrhage, (3) wounds of the stomach or intestines large enough to permit extravasation.

Dr. McRae quoted from contributions to this subject by Senn, Nunez, Winslow, Budinger, Vaughan, Woolsay, Robson, Chauvel, Michaux, Klemm, etc., and said, from the conflict of opinion and advice given by these and various other surgeons, one is at a loss to know just how to proceed in many cases. His own convictions, based on sixteen cases which he narrated, are positive. While he has the greatest admiration and respect for the surgical opinions of Senn, he should be recreant to his convictions were he to accept the proposition of non-interference laid down by Senn in his recent contributions to the subject. In most of the cases which have come under his observation, where grave lesions of the abdominal viscera have existed, the symptoms of internal injury were practically *nil*, and the positive signs of grave damage almost entirely absent.

In conclusion, he reiterated his firm conviction that every case of penetrating wound of the abdomen, where there is reasonable doubt as to the injury of the abdominal viscera, should be subjected to at least an exploratory operation. This enables the surgeon to either be absolutely sure that no injury has been done to the viscera, or to repair, as far as possible, such injuries when they exist. The mortality following a simple exploratory operation, *per se*, is so small as not to weigh against the much greater mortality that necessarily follows the expectant plan of treatment. In the cases he had seen, the general and local signs have been so misleading and enigmatical that, prior to the operation, a conclusion on his part as to internal conditions would have been entirely guesswork.

### THE PRACTICAL MANAGEMENT OF BULLET-WOUNDS OF THE ABDOMINAL VISCERA.

This paper was read by Dr. Horace H. Grant, of Louisville, Ky. Observation and experience with operators coming frequently in contact with this lesion have determined that penetrating wounds of the abdomen demand laparotomy at the earliest possible moment after diagnosis and thorough inspection of the entire region endangered. It is established that not only is the mortality in untreated wounds of the abdomen almost one hundred per cent., where the intestines are perforated, and very high in wounds of the solid viscera, but it becomes less promising every advancing hour, until by the second day peritonitis sets in and destroys the hopefulness of the prospect. Within the past two years the speaker has operated upon four cases. In each case the patient was seen early, in no case later than four hours. In three, resection of the intestine was necessary. Three of these patients recovered; the fourth died from septic peritonitis, due to the escape of a large quantity of fecal matter before operation. Autopsy disclosed a perfect intestine.

These three papers were discussed together.

Dr. W. L. Robinson, of Danville, Va., said he has seen a good many cases of gunshot wounds of the abdomen, and nearly all of them died, either with or without operation. Early operative interference might save some of them.

Dr. G. S. Brown, of Birmingham, said he knew of several cases of gunshot wounds of the abdomen that occurred in and around Birmingham, that recovered without operation. Personally he had operated on five cases, one of which recovered. In this case there was no perforation of the intestine. Undoubtedly, diarrhea and starvation, mentioned by Dr. Parker, in his paper, have something to do with the favorable results. The intestines are most liable to be perforated when full.

Dr. Willis F. Westmoreland, of Atlanta, said the results of gunshot wounds of the abdomen on the battle-field should not be compared with those met with in civil life. It is not a question of operating, so far as fatal results are concerned, but the manner in which the operation is done.

Dr. W. E. B. Davis, of Birmingham, thought that Dr. Parker's paper was not an argument against operative procedures for abdominal wounds. Surgeons on the battle-field do not have the same facilities for operating on penetrating wounds of the abdomen that they do in civil practice, and which are necessary for the proper care of cases. If a man sustains an injury to the intestine, if his abdomen is not opened, death may be expected. In twenty or more cases operated on by him and his brother, all of the cases died that were seen twenty-four hours after the injury. This is not an argument against operative interference, but against late operation. Statistics impress surgeons with the importance of early operation.

Dr. George A. Baxter, of Chattanooga, believes it is not necessary in many instances to open the abdomen in cases of gunshot wounds, and cited a number of cases that recovered without operative interference.

Dr. John T. Wilson, of Sherman, Texas, advocated early operation in cases where the surgeon is convinced that perforation has taken place. The character, technique and rapidity of the operation are very important. In operating, the intestines should be exposed as little as possible.

Dr. W. L. Rodman, of Louisville, said that while he had pursued the practice of operating on penetrating gunshot wounds of the abdomen for the last ten years, after hearing the paper of Dr. Parker and reading the recent contributions of Senn and others, he thought whether he as well as other surgeons may not have gone too far in operating on so many of these cases; at the same time, he does not wish to be understood as advocating a masterly inactivity where the environments for operative interference are favorable.

Dr. Westmoreland said that given a case of perforation of the intestine, particularly where there is extravasation of contents, instead of being masterly inactivity it is criminal inactivity for a surgeon not to operate.

Dr. Parker, in closing the discussion on his part, said that he had tried to make a clear distinction between the plan of treatment of gunshot wounds of the abdomen met with on the battle-field and those encountered in civil life. He is strongly opposed to laparotomy on the battle-field on account of the conditions that exist. In civil life, however, the thing to do in cases of gunshot wounds of the abdomen, is to got in and out of the belly as soon as possible consistent with good work.

Dr. McRae believes that a large proportion of the operations undertaken for gunshot wounds of the abdomen late will terminate fatally.

Dr. Grant stated that practically all cases operated on after twenty-four hours perish. It is impossible to diagnosticate perforation of the intestines with any degree of certainty. If a patient is seen within eight or ten hours after the receipt of a gunshot wound of the abdomen, under favorable surroundings, whether the bullet has penetrated or not, the skilful surgeon will not hesitate to make at least an exploratory operation, because it would not hurt the patient, but would probably save his life.

### THE TREATMENT OF COMPLETE RUPTURE OF THE PERINEUM BY DISSECTING OUT THE SPHINCTER MUSCLE AND ITS DIRECT UNION BY BURIED SUTURES.

Dr. Howard A. Kelly, of Baltimore, Md., read a paper on this subject, in which he said the results of the best methods of the treatment of complete tears of the perineum are not entirely satisfactory in a large percentage of cases. The control over liquid motions and flatus is, as a rule, not secured immediately, and it is usually necessary to encourage the patient by telling her that she "will have to learn to control the muscle in the course of time." Such a control, more or less perfect, is gained in course of several months. This defect in our present procedures is due to a faulty approximation of the sphincter ends which lie buried in a pit, and are therefore difficult to bring into accurate, firm apposition by sutures embracing a considerable quantity of tissue surrounding the sphincter ends. Dr. Kelly proposes therefore the deliberate dissection and freeing of the sphincter ends, drawing them out about one and a half centimeters from the tissues, cutting off the scarred ends, and effecting a direct union of the freshened ends by two or three buried catgut sutures. He was led to do this operation by his experience in a case which had been operated upon six times with a result, which, judged by superficial appearances, was perfect, and yet the patient had no control over her bowel functions. He made a semi-lunar incision around the anterior periphery of the anus and found the right sphincter end buried in scar tissue in the median line, while that of the left side was ectopic and attached out under the ischial tuberosity. The sphincter ends were united directly by buried catgut sutures, and the skin wound closed and union took place *per primam.* In addition to these buried catgut sutures a splinting suture of silkworm gut is passed through the middle of the sphincter near the edges of the wound and on up through the septum splinting the ends together and taking the tension off the catgut. He has since taken the hint given by this case and adopted a similar procedure in six cases of complete tear of the perineum due to confinement. Two additional cases were operated upon by Dr. W. W. Russell and one by Dr. Ramsey. In each instance there was a surprising difference between the new and older methods, noted at once in the earliest stages of the convalescence, and the patient was immediately conscious of perfect control of her functions. The bowels should never be locked up.

Great care must be taken not to leave any dead spaces in closing the remainder of the wound in the usual way, in order to avoid all risk of infecting the buried sutures. He only recommends this operation to those who possess considerable skill in doing plastic operations and in securing a snug, accurate adaptation of the parts.

In the discussion, Drs. George H. Noble, of Atlanta, and J. Wesley Bovee, of Washington, D. C., added their testimony to the usefulness of the method described by Dr. Kelly, but certain features of the technique of which they modified.

Dr. Lewis S. McMurtry, of Louisville, Ky., read a paper on "The Treatment of Cancer of the Uterus."

DISCUSSION.

Dr. William L. Rodman, of Louisville, asked the members to give their experience relative to the frequency of cancer in the black and white races. He was rather surprised to hear of the infrequency of the disease in the negro woman. According to the last census statistics of Billings, cancer of the uterus is more common at all ages in the black than in the white race. This is also the experience of Matas, who has examined the records at the Charity Hospital in New Orleans. An examination of all deaths recorded by the Health Department at Louisville for the past thirty years corroborated the same view.

Dr. Ernest S. Lewis, of New Orleans, stated that, while he had not observed a very marked difference in the relative immunity of negro women to cancer of the uterus, still, if his experience is not at fault, he thinks, owing to their uncleanliness, their mode of living, and to the more frequent accidents to which they are subject during labor, they are particularly prone to cancer of the uterus. With regard to the results of all operations for cancer of the uterus, he indorsed everything Dr. McMurtry had said. He could only recall one case of vaginal hysterectomy for cancer, that lived for eight years, after which the disease returned, and the patient finally died. The disease is so liable to return that he considers any operation as palliative, and he believes that would be the result of the abdominal operation advocated by Dr. McMurtry, particularly in advance cases; and if cases are met with in the incipiency of the disease, in his opinion as much could be accomplished by the vaginal as by the abdominal operation.

Dr. Virgil O. Hardon, of Atlanta, said that during his seven years' connection with the Grady Hospital, he had reason to believe that cancer of the uterus was more frequent in negro women than in white women. He has been led by his experience in the treatment of uterine cancer to the same conclusion as that reached by the essayist, except he had been led to go further. He has operated for cancer of the womb by vaginal extirpation, by the abdominal method, and by the combined method, and he has never yet operated upon a case where recurrence did not take place sooner or later, and for this reason he has lost all confidence in operative measures as a means of effecting a permanent cure. However, he has no doubt, on theoretical grounds, that if cases are seen sufficiently early, a permanent cure might be effected by surgical interference.

Dr. Howard A. Kelly, of Baltimore, remarked that he was astounded at the trend the discussion had taken, because he had seen dozens of cases of cancer of the uterus that had remained well for a number of years after having undergone surgical intervention, removing the uterus either by vagina or by the abdomen. He finds carcinoma of the uterus as frequently in negroes as in white women.

Dr. J. Wesley, Bovee, of Washington, D. C., said that his experience in the radical treatment of cancer of the uterus has been more satisfactory than what he had been hearing to-day. He knows of a good many cases upon which he has operated that have gone on for three years or more without a recurrence of the disease. He has done three operations after the manner described by Werder in the *American Journal of Obstetrics* of last winter, and he is much pleased. In each case he adopted the abdominal rather than the vaginal route, believing a more radical operation could be performed by this method. He has great hopes for the future treatment of cancer of the uterus by complete abdominal hysterectomy, undertaken early.

Dr. William L. Robinson, of Danville, said that when the parts have become infected beyond the uterus, no dissection, however extensive, would ever remove the cause. When the disease has extended beyond the uterus, he believes it is beyond the power of any surgeon to thoroughly remove it. Therefore, unless an operation could be done very early, he would not advise the removal of the uterus.

Dr. W. E. B. Davis was profoundly impressed with the position taken by Dr. Lewis as to the ultimate outcome of cases of cancer of the uterus, believing his position is correct. There is no man in America who has had a larger experience in the treatment of cancer of the uterus than Dr. Lewis, of New Orleans; and the profession were familiar with his great skill as a surgeon, hence his experience regarding uterine cancer was certainly valuable.

Dr. McMurtry, in closing the discussion, said he could not indorse the rosy view that Dr. Kelly takes with reference to the ultimate results of the operative treatment of cancer of the uterus. He knows that Kelly's work in this direction has been extensive and that his reports are sincere and reliable. But there are a great many surgeons, working in the same line, who have done thorough, faithful, skillful work in the radical treatment of uterine cancer, who doubt the future confirmation of Dr. Kelly's views.

PRESIDENT'S ADDRESS.

This was delivered by Dr. Richard Douglas, of Nashville, Tenn. He made a departure from the usual or stereotyped addresses, and selected for his subject Acute General Peritonitis. Before considering his subject proper, he spoke of the growth of the Association and the excellence of its scientific work, saying that the Association owes its existence and high standing to the indefatigable efforts of Dr. W. E. B. Davis, the permanent secretary.

A bacteriological classification of peritonitis is beset with many difficulties, and while the speaker is free to admit that for all practical purposes peritonitis is of bacterial origin, yet there occurs a respectable percentage of cases in which the most rigid examination fails to disclose the presence of micro-organisms. Hartmann and Moreau report in detail such a case recently. Of one hundred and ten cases bacteriologically classified by Flexner, there were twelve cases which he was obliged to consign to the idiopathic group. It is true that in all of these patients there existed conditions predisposing to peritonitis, such as cardiac, renal or hepatic disease. In the intensely septic mycotic form of peritonitis their absence may be accounted for by the fulminant type of the disease, death occurring from intoxication before the colonies of bacteria are well established. Any effort to individualize the effect of a specific bacterium, to connect its presence with a definite pathological phenomenon and characteristic symptomatology is as yet futile. Mikulicz avers that all forms of peritonitis run the same clinical course regardless of the bacteria that cause it. There is a law laid down by Maloz to the effect that if the peritonitis is of intestinal origin, the colon bacillus will show it; if it is of uterine origin, that is, connected with abortions or labor, we will find the streptococcus. It is asserted that the colon bacillus constitutes nine-tenths of the bacteria of the digestive tract, the colon is its natural habitat, but its behavior upon gaining entrance to the peritoneal cavity depends upon many circumstances, and this bacillus has many morphological conditions, many forms and stages. The constancy of its presence in peritonitis, since the demonstrations of Welch and others of its capacity of transmigration through the bowel, has led many to attach to it great significance as an etiological factor, but its association with other forms of micro-organisms renders the first proposition of Maloz null and void. If we interpret Maloz's law correctly, the absence of streptococci would eliminate the possibility of peritonitis being of puerperal origin. In rebuttal of this idea, Dr. Douglas quoted from Winckel, who says, "Kroenig examined all parts of the uterus of a woman dead from infection and found not only the superficial thrombi at the placental site thickly filled with bacteria, but also the serous lining of the peritoneum." This case shows that a woman may have a peritonitis following abortion without streptococci, and it further shows that the colon bacillus without an intestinal lesion may produce a fatal peritonitis. No doubt too much importance has been ascribed to the colon bacillus, and it is interesting to note that Tavel, who was among the first to emphasize the pathogenic powers of the bacillus coli communis, thinks now the bacillus of minor importance in the etiology of peritonitis. He holds further that the very name of the bacillus coli should be considered merely a collective name of many varieties of bacteria.

No better argument against bacteriological classification can be adduced than the words of Simon Flexner, who says: "In order that pathogenic bacteria introduced directly into the peritoneal cavity may cause a peritonitis, general or circumscribed, evanescent or fatal, the normal conditions of the peritoneum must in some way be modified." It is clear, then, that in the human being, as in experimental animals, some other condition than the mere presence of pathogenic micro-organisms in the abdominal cavity is necessary in order that peritonitis may be produced. Bacteria alone and unaided by physical conditions are comparatively innocuous.

Restricting consideration entirely to acute general peritonitis, it occurs from traumatic or consecutive causes. Under the head of traumatic peritonitis is embraced all infective inflammations arising from wounds of the peritoneum, whether accidental or operative, penetrating or non-penetrating. Consecutive, secondary or symptomatic peritonitis may be subdivided into (1) peritonitis by continuity, and (2) perforative peritonitis.

*Traumatic Peritonitis.* Under this head all forms of peritonitis must be embraced that are due primarily to a trauma. The destruction of tissue, the retention of blood-clots and natural secretions, prepares a suitable soil for microbic invasion, and this essential infecting element may be introduced from without as through penetrating gunshot, stab, or operative wounds, under which circumstances it is usually a poly-infection, the streptococcus predominating. There are also contusions of the abdomen without an external wound which so injure and lower the resistance of the tissue as to favor the migration of the intestinal micro-organisms and their infection of the general cavity.

*Secondary Peritonitis.* The supervention of acute general peritonitis, as secondary to preexisting disease processes, embraces in its etiology infective inflammation not only of all intraperitoneal viscera, but all organs or tissues contiguous thereto, and it does not appear to him to be too broad if there be included within its scope those peritonitides of hematogenic or metastatic origin, for it is well established that the specific germs of diphtheria, scarlet fever, pneumonia and erysipelas have produced acute general peritonitis.

Peritonitis by continuity is the uninterrupted extension of the inflammation from an infected area to the peritoneum, and is accomplished by the invading army of micro-organisms through the lymphatics, blood channels, or by direct penetration of tissues. The reactionary inflammation which determines the localization or diffusion depends upon the virulence of the bacteria, the resistance of the tissues, and, what is too frequently overlooked, the individual resistance of the patient. Puerperal peritonitis of streptococcus origin conspicuously illustrates inflammation by continuity.

Perforative peritonitis is the next subdivision of secondary peritonitis. The sudden opening of a focus of suppuration and the discharge of its contents into the peritoneal cavity, whether it be from hepatic abscess, a pus tube or mesenteric gland, is a true illustration of perforative peritonitis; yet, the inflammation and systemic infection following is not necessarily so conspicuous. The duration of the primary disease, the virulence of the micro-organisms, the preparedness of the peritoneum by fortifications of adhesions, the general condition of the patient, are all circumstances which may modify and circumscribe the peritonitis. It is more common for perforative peritonitis to occur as a result of ulceration of the wall of some of the hollow viscera as in peptic ulcer, typhoid and dysenteric intestinal ulceration, duodenal and appendicular ulceration.

The conclusions that may be drawn from an etiological study of peritonitis may be thus summarized: Traumatic peritonitis, especially the post-operative variety, is essentially a grave condition, not only because there is immediate or primary inoculation of the peritoneum, but the conditions are all favorable for germ culture and dissemination. Peritonitis by continuity may become general and prove rapidly fatal, but this is not the rule except in puerperal cases. Contrary to the expressed opinion of more than one writer, there is nothing peculiar about the peritoneum, or the cecum, or appendix, or the true pelvis which accounts for the more frequent localization of inflammation in these regions than in other areas of the abdomen. The method of invasion, the activity of the process and resistance of patient alone determine the local or general type of peritonitis. Visceral perforation, whether traumatic or pathological, is an ideal condition for germ culture and the elaboration of toxins. Their rapid absorption and general diffusion throughout the peritoneum sufficiently explain the grave state into which the patient is precipitated. It may be asserted that the clinical course and pathological expression depend largely upon the nature of the exciting cause, the character of the preexisting disease or injury, and the mode of invasion.

Without attempting to formulate any definite pathological classification of general peritonitis, and to adapt each to its special cause, he prefers to direct his efforts to simplifying and dispelling the confusion that exists. He accepts, with slight reservation, the now almost universally conceded idea that acute general peritonitis is and must be septic, that is, of bacterial origin. But he maintains that surgeons do not understand each other, nor have they all a clear conception of what is meant by septic peritonitis. It has been a race for life between the practical surgeon and bacteriologists as to who should claim the honor of naming the pathological phenomena in this great serous bursa. He maintains that the surgeon is alone competent to define, classify and prognosticate the protean types of this disease. That men are honest, painstaking and accurate goes without saying, yet how can surgeons reconcile the report of McCosh's last series of eight cases of general septic peritonitis and six operative recoveries with the experience of Senn "of many cases of diffuse septic peritonitis without a single successful result?" The answer certainly is not in the superior skill or special technique of the operator, but it is to be found in an analytical study of McCosh's cases; six of them were purulent peritonitis and two serous peritonitis, not the class of cases referred by Senn at all. Tietze defines diffuse septic peritonitis to be "that form of peritonitis in which there is little or no exudate, severe symptoms of intoxication and terminating rapidly fatally."

Some four years ago, Dr. Douglas reported eight cases of general peritonitis subjected to operation, with four recoveries and four deaths. He dealt with them all as cases of general septic peritonitis from a misconception of the term. Two of them only properly belonged to this class and they terminated fatally. The others were cases of general purulent peritonitis, with two deaths and four recoveries. He is very well pleased with his percentage of recoveries yet, but disgusted with his classification. He did not discriminate between diffuse septic peritonitis, the patients dying in twelve hours with profound toxemia and dry peritoneum after perforation of appendix, and one of perforation with enormous purulent effusion, but mild symptoms of sepsis. This error of his is the common one with the profession, and it is the outcome of an attempt to classify peritonitis by the character of exudate.

The etiological classification of peritonitis places it as subsidiary to the primary lesion that produces it, and its practical import is its local origin. A painstaking investigation into the clinical history will usually determine the origin or structure primarily involved. The symptoms and physical signs, if the case is seen sufficiently early, will still further aid in incriminating the part under suspicion. This valuable diagnostic evidence does not conform to any stereotyped expression; the symptomatology of peritonitis is as varied as its causes. By the presence of any striking symptom, or group of symptoms, physicians cannot arrive at a decision in a doubtful case, but from the whole picture they must form conclusions.

Dr. George H. Noble, of Atlanta, read a paper on

URETERAL ANASTOMOSIS.

He gave an analysis of thirty cases collected from literature, and divided them into two classes: First, ureteral anastomosis, or union of the ureter end to end or end to side; second, ureteral implantation into the bladder. July 2, 1896, the essayist ligated the ureter with the ovarian vessels and Fallopian tubes in a case of large extraperitoneal fibroma. This was the very first ligature applied in doing the hysterectomy. The bladder end was split about three-eighths of an inch, as much of the mucous membrane as could be drawn out was cut away; then the upper, or kidney end, of the ureter was invaginated into it, using a mattress suture for the purpose. The split was then closed with fine sutures, making a snugly-fitting cuff around the opposing end, with no mucosa intervening. Small silk was used to stitch the raw edge of the inferior segment around and to the sides of the upper section. A peritoneal cuff was thrown around the joint thus made and the ureter buried behind the peritoneum. The entire work was conducted with the aid of a ureteral catheter, which had been introduced through the ureter into the bladder and drawn out of the urethra with a pair of forceps. The upper end was passed into the kidney end of the ureter. This served a useful purpose, the entire manipulation being greatly facilitated and preventing obstruction of the ureter by crowding down the cut end of the mucosa just below the line of union. This case had suffered from absorption of pus for a number of weeks from double pyosalpinx, was emaciated and thoroughly septic. Her temperature just before going upon the operating-table was 103°, and had been running equally as high for the time she had been in the hospital, and perhaps longer. Her pulse was 130 per minute, weak and thready. Thus this anastomosis was done under unfavorable conditions, but was successful.

In October, 1898, he did a successful resection of the left ureter in a case of extraperitoneal extension of sarcoma of the ovary. About one and a half inches of the duct were removed and ends united as above. The peritoneum was stripped from the entire left half of the pelvis. The bladder was separated from the upper portion of the vagina, and also from the horizontal ramus of the pubic bone, and drawn up to left side to the ileo-pectineal line to cover or bury the ureter, and fastened with a silk suture. In this instance the ureteral catheter was introduced on the stilette, and being stiffened by it passed directly out of the urethra from the ureter; thus the use of forceps was necessary to fish it out of the bladder. Abdominal and vaginal gauze drains were used. There was no leakage.

Dr. Noble said that there are several features necessary to success in doing ureteral anastomosis aside from thorough asepsis. (1) Perfect coaptation or invagination. This must be done in such a way that no mucous membrane can slip between the two ends of the ureter. (2) The ureteral artery should be preserved in its continuity to the ends of the ureter. If this vessel be destroyed when the ureter has been dissected loose from its attachments, it will perish beyond the point where it ceases to receive its blood supply. The ends of the ureter should be cut back to the point where the artery is intact lest under diminished blood supply and tension it should perish. All strain or tension must be relieved. Should union occur by first intention, the parts will separate as soon as the sutures cut out or are released. (2) There must be no obstruction to the flow of urine.

### CARCINOMA OF THE BREAST.

Dr. Willis F. Westmoreland, of Atlanta, read a paper on this subject, in which he said that carcinoma now presents even a more serious problem than formerly, on account of its rapid increase. The proportionate mortality from cancer is four and a half times greater now than half a century ago. No other disease can show anything like such an immense increase. According to statistics, two out of every five cases of carcinoma in the female are of the breast. Over three-fourths of all the tumors occurring in the breast are carcinoma. Or, to be exact, of 637 tumors, over 503, or 83.20 per cent., were carcinoma, leaving only 107, or 16.79 per cent., representing all other forms of neoplasms. The author discusses the etiology of the disease at considerable length. Speaking of the pathology, he said there was no subject in surgery about which such divergent opinions are held. There is a wide variation in the malignancy of carcinoma, some proving fatal in less than a year, a few others taking ten or fifteen years. He divided the varieties of carcinoma into scirrhous and medullary or encephaloid. The former has an average duration of life of about thirty months, the latter of about twelve months. When it is considered that the skin and pectoral muscles are involved in nearly every instance in this disease, the surgeon can easily see the philosophic reasoning upon which Halstead's operation is based. The essayist follows the Halstead method in carcinoma of the breast. He believes, however, Dr. Bloodgood was the first to demonstrate the advantages of completely cleaning out the postero-internal sub-scapular region by the supra-clavicular route. The results of the Halstead operation, in the opinion of the essayist, depend upon the ability of the individual operator, his earliness in seeing the case, and his closeness in following Halstead's technique. This operation not only requires surgical skill and experience, but a proper respect for the magnitude of small things. In the opinion of Dr. Westmoreland, no surgeon will make a satisfactory operation on his first few cases. He finds that with each case he operates more satisfactorily. The operation does not present the seriousness that its magnitude would lead us to believe. He has operated six times before his class at a public clinic, and transferred the patients afterwards, at times quite a distance, to their homes. In his first operations he had some shock from exposure, but this is easily controlled by placing hot towels over the exposed surfaces. There is practically no blood lost in a properly made operation. It is better to complete the operation, grafting included, at one sitting. When, for any reason, this is not practicable, the cleaning out of the supra-clavicular region and the grafting can be made at a second operation. When grafting is done at a second operation he finds it better to remove the granulations. This is best done by curetting. In two of his cases it was necessary to remove the sub-scapular nerves, and in another to resect the axillary vein. No unpleasant sequela followed in either case. He has operated upon over fifty cases of carcinoma since he began surgical work, and he has always cleaned out the axilla and stripped the muscles in each case. He has been making Halstead's operation since its introduction. He does not know the results in the majority of these cases. Two of them, however, came back to him with later recurrence, one with a carcinoma of the femur five years after operation, no local return; the other with a local return in the cicatrix nine years afterwards. This case died in a short time from metastasis. He feels quite sure that these were not independent new growths. There is always one rule, that the more malignant the case the greater the blood changes.

The primary object of the paper was to point out the fact that a careful clinical examination of each case, a record of the local conditions, the type of tumor, the blood changes, whether or not there was marrow infection, the family history, and the length of time it had existed, would not only give definite facts upon which to base a prognosis, but, if published with each case, would give all surgeons a record of infinite value for comparative purposes.

The following officers were elected for the ensuing year:

*President*—Dr. Joseph Taber Johnson, of Washington, D. C.

*Vice-Presidents*—Drs. F. W. Parham, New Orleans, and W. L. Robinson, Danville, Va.

*Secretary*—Dr. W. E. B. Davis, Birmingham, Ala.

*Treasurer*—Dr. A. M. Cartledge, Louisville, Ky.

*To fill vacancy in the Council*—Dr. L. McLane Tiffany, Baltimore, Md.

Place of meeting, New Orleans; time, to be determined by the Secretary with advice of the Council.

After the presentation and adoption of resolutions of thanks, the Association adjourned.

---

### THE TREATMENT OF FRACTURES.

Dr. W. L. Estes (*International Journal of Surgery*) says:

First. Unless a fragment is threatening to break through the skin it should never be reduced except by the physician, and then only when the parts can be kept in permanent apposition.

Second. Men carrying an injured person should not keep step, as the jar is greater.

Third. Strychnia should be given for shock, morphia for pain, but no alcohol.

It is very rarely necessary to make a patient go through the double agony of temporary and permanent setting of the broken bones.

In simple fracture gentle rubbing of the ends will assist in getting rid of shreds of tissue which are invariably caught there.

Nowadays a surgeon will rarely be satisfied that a bone is properly set until verified by the X-rays.

Plastic splints, preferably plaster of Paris, are the best apparatus.

No simple fractures require constant confinement to bed, except of the innominata and upper third of the femur.

Do not wait for the swelling to disappear before putting on a permanent dressing.

A well-applied splint with good apposition of the fragments should not be removed too early. Proper time for massage is two or three weeks after fracture of upper extremities and four or five weeks for lower extremities.

---

**NOTICE.—This JOURNAL and THE INTERNATIONAL JOURNAL OF SURGERY one year for $2.00 cash.**
